# An Adaptogen: Withaferin A Ameliorates *in Vitro* and *in Vivo* Pulmonary Fibrosis by Modulating the Interplay of Fibrotic, Matricelluar Proteins, and Cytokines

**DOI:** 10.3389/fphar.2018.00248

**Published:** 2018-03-22

**Authors:** Swarna Bale, Pooladanda Venkatesh, Manoj Sunkoju, Chandraiah Godugu

**Affiliations:** Department of Regulatory Toxicology, National Institute of Pharmaceutical Education and Research, Hyderabad, India

**Keywords:** Withaferin A, pulmonary fibrosis, epithelial to mesenchymal transition, extracellular matrix, inflammation, angiogenesis

## Abstract

Pulmonary fibrosis (PF) is chronic lung disease with only two FDA approved clinically available drugs, with limited safety profile. Inadequate therapy motivated us to explore the effect of vimentin inhibitor Withaferin A, as an anti-fibrotic agent against TGF-β1-induced *in vitro* fibrotic events and Bleomycin induced *in vivo* fibrosis with an emphasis on epithelial to mesenchymal transition (EMT), extracellular matrix deposition (ECM), inflammation, and angiogenesis. *In vitro* EMT and fibrotic events were induced by TGF-β1 in alveolar epithelial cells and human fetal lung fibroblasts followed by treatment with Withaferin A (0.25, 0.5, and 1 μM concentrations) to explore its anti-fibrotic effects. *In vivo* potential of Withaferin A (2 and 4 mg/kg) was assessed in murine model of Bleomycin induced PF. All the parameters and molecular studies related to PF were performed at the end of treatment period. Withaferin A treatment reduced the progression of PF by modulating the EMT related cell markers both *in vivo* and *in vitro.* Withaferin A ameliorated the expression of inflammatory cytokines including NF-κB p65, IL-1β and TNF-α, as well as attenuated the expression of pro-fibrotic proteins including CTGF, collagen 1A2, collagen 3A1, and fibronectin. Expression of angiogenic factors like VEGF, FAK, p38 MAPK, and PLC-γ1 were also inhibited by Withaferin A. Phosphorylation of Smad 2/3 induced by TGF-β1 and Bleomycin were significantly inhibited. Withaferin A suppressed expression of pro-inflammatory, pro-fibrotic, and pro-angiogenic mediators and also reduced the ECM deposition. In a nutshell, Withaferin A could probably prove as an efficient and potential therapeutic against PF.

## Introduction

Pulmonary fibrosis (PF) is a chronic, progressive and irreversible lethal medical problem hallmarked by alveolar disruption, excess scarring and edema of lungs that may significantly contribute to critical respiratory failure (American Thoracic Society and European Respiratory Society, 2002; King et al., 2011). PF is common amongst various interstitial lung diseases with poor average reported survival rate of 2–3 years (Ley et al., 2011). Etiological factors of PF are not well-established and some of the reported findings promoting PF include exposure to chemotherapeutics and other xenobiotics such as silica, asbestos, and paraquat (Wilson and Wynn, 2009). Persisting respiratory disorders like asthma, chronic obstructive pulmonary disease, severe lung injury, environmental factors, and uncontrolled wound healing also culminate in the progression of PF (Chilosi et al., 2012). Though noticeable efforts have been consistently made to develop novel therapeutic strategies for treatment of PF including steroids and immunosuppressant agents, they could not sufficiently intervene the fibrotic progression and thus are considered only as supportive therapies. Esbriet (Perfenidone), a potential TGF-β inhibitor and Ofev (Nintedanib), a tyrosine kinase inhibitor are the only two USFDA approved drugs available to treat PF till date (Ley et al., 2011). Unfortunately, these drugs could not satisfactorily overcome the progression of disease and moreover they exhibit gastrointestinal, skin, and liver-related adverse effects (Jiang et al., 2012; Richeldi et al., 2015). Despite the advances in the arena of PF, the urge to develop efficient therapeutics prevails.

Withaferin A is a natural steroidal lactone, rejuvenating tonifier, immunomodulator and a pre-dominant bioactive constituent obtained from *Withania somnifera* (Ashwagandha) which exhibits an array of potential biological activities including anti-inflammatory, anti-invasive, pro-apoptotic, and anti-fibrotic effects and is remarkably safe (Vanden Berghe et al., 2012; Hahm et al., 2014; Madhusudan et al., 2016; Kim et al., 2017). WFA exhibits potent anti-inflammatory effect by downregulating central inflammatory mediator, nuclear factor kappa light chain enhancer of activated B cells (NF-κB) and other cytokines which has been well-elucidated *in vitro* and *in vivo* studies (Heyninck et al., 2014). Above all, WFA is a pre-clinically proven vimentin and TGF-β inhibitor but its role in PF is not yet explored (Challa et al., 2012). Thus, the present study is aimed at demonstrating the role of WFA in mitigating PF.

Epithelial to mesenchymal transition and extracellular matrix (ECM) are considered as crucial developmental milestones in PF wherein, a pivotal fibrogenic cytokine TGF-β is aberrantly expressed, which in turn triggers EMT process thereby enhancing ECM deposition mediated by both Smad dependent and independent pathways (Verrecchia and Mauviel, 2002; Kasai et al., 2005; Kolosova et al., 2011; Todd et al., 2012; Zhang, 2017). Though decisive factors of PF are questionable, some of the mechanisms that have been found to be imperative in disease progression include inflammation, oxidative stress, deregulated ECM and EMT signaling. Therefore, targeting these pathways may lead to discovery of potent novel compounds with anti-fibrotic activity, thus diminishing the existing void in treatment of PF. In light of key evidences of WFA as a vimentin, TGF-β and NF-κB modulator, the present study investigates the potential of WFA in ameliorating PF with an emphasis on matricellular and fibrotic proteins. Mechanisms of pharmacological intervention by WFA were evaluated through molecular techniques like immunohistochemistry, immunocytochemistry, ELISA, and western blotting.

## Materials and Methods

### Reagents and Antibodies

Withaferin A was procured from Aptus laboratories (Hyderabad, India) and TGF-β1 was obtained from Bio-legend (United States); Bleomycin sulfate was procured from Cipla labs (India); Masson’s trichrome staining kit, Sirius red, Chloramine-T, Hydroxyproline, and Ehrlich reagent were procured from Sigma-Aldrich, Anti-ZO-1, anti-E-cadherin, anti-Smad 2/3, anti-p Smad 2/3, anti-vimentin, anti-NF-κB p65, anti-p VEGF, anti-p p38 MAPK, anti-p FAK, and anti-p PLCγ1 were procured from Cell Signaling Technology, while anti-Col 1A2, anti-Col 3A1, anti-smooth muscle actin, anti-CTGF, anti-fibronectin, and anti-TGF-β1 were obtained from Santa Cruz Biotechnology (United States). ELISA kits were purchased from eBioscience, United States. TGF-β bioplex kit was procured from Merck-Millipore. Rest of the chemicals and reagents were of analytical grade and obtained from commercially available sources.

### Cell Culture

HFL1 cells were procured from ATCC (ATCC^®^ CCL153^TM^) and A549 cells were purchased from National Centre for Cell Science (NCCS, Pune, India). HFL1 and A549 cells were cultured in F-12K medium (ATCC) and RPMI medium (Sigma-Aldrich, United States) respectively; supplemented with 10% fetal bovine serum and 1% anti-biotic solution (Invitrogen, United States). TGF-β1 was selected to induce fibrotic events at a concentration of 10 ng/mL in both HFL1 and A549 cell lines. WFA was dissolved in DMSO and a stock concentration of 10 mM was prepared, stored at -20°C and diluted with respective media at required concentrations before use. Cells were cultured and treated with WFA at various concentrations (0.25, 0.5, and 1 μM) 2 h prior to induction of TGF-β1 and incubated for 24 h at 37°C maintained in 5% CO_2_ incubator. All the experiments were performed in three internal replicates.

### Cell Viability Assay

The effect of WFA on viability of HFL1 and A549 cells was evaluated using MTT assay (Maurya et al., 2011). Briefly, 10,000 cells per well of HFL1 and A549 were seeded in 96-well plates and after 24 h of incubation, WFA was treated at concentrations in serial dilution from 0.78 to 50 μM (7 serial dilutions). Post 24 h of incubation, MTT solution was added at a final concentration of 0.5 mg/mL, incubated for 4 h followed by addition of DMSO to dissolve the formazan crystals then incubated for 30 min and absorbance was measured at 570 nm with a multi-mode spectrophotometer. Experiments were performed thrice in triplicates and data was expressed as percentage cell viability versus concentration of the compound by taking control cells as 100% viable cells.

### Cell Migration Assay

The ability of the cells to migrate was assessed using cell migration assay. Briefly, HFL1 cells were plated at a density of 1 × 10^5^ cells in 12 well culture plates and allowed to grow till 70–80% confluence was attained. The monolayer of the cells was disrupted to create a gap in a straight line using a sterile 200 μL micropipette tip, and the wells were washed with PBS to remove the detached cells (Choi and Kim, 2015). Cells were treated with TGF-β1 and WFA for 24 h. Images of the gap between cells were captured at time points of 0 and 24 h, the width of the gap was measured in all groups using microscopic scaling and quantified using image J software.

### Immunocytochemistry

Cells were cultured on cover slips and after 24 h of plating, cells underwent starvation (media with no serum) for 6 h, followed by WFA treatment 2 h before TGF-β induction. After 24 h of incubation, cells were fixed with 4% paraformaldehyde for 15 min, washed with PBS and permeabilized with 0.1% Triton X-100 in PBS for about 30 min. Cells were washed twice with PBS and incubated for 1 h with blocking solution (3% BSA in PBS). The blocked cells were incubated with primary antibodies anti-vimentin and anti-α SMA at 1:100 dilutions in 3% BSA overnight at 4°C. On the following day, cells were washed thrice with PBS, incubated with secondary Fluorescein isothiocyanate (FITC) anti-rabbit and rhodamine anti-mouse antibodies for 2 h at room temperature. Cells were washed thrice in PBS (5 min per wash) and then cover slips were mounted with vectashield hardset antifade mounting medium^®^ with DAPI (Vector Labs, Burlingame, CA, United States) on a glass slide. Slides were visualized using a confocal microscope (Leica TCS SP8 Laser Scanning Spectral Confocal).

### TGF-β Signaling 6-Plex Magnetic Bead Assay

Stimulated and unstimulated cell tissue lysate were used to detect changes in phosphorylated Smad2 (Ser465/Ser467) and Smad3 (Ser423/Ser425) using the Luminex system. This assay was performed with customized highly sensitive MILLIPLEXMAP signaling 6-plex magnetic bead human TGF-β kit (Cat # 48-614MAG Millipore) based on protocol provided by manufacturer.

### Animals and Treatment

Animal studies were carried out in male Swiss albino mice, 8 weeks old, purchased from Teena labs (Hyderabad, India) and acclimatized for a week before study initiation. Animals were allowed free access to food and water *ad libitium* and maintained with 12/12 h light and dark cycle. All the animal experiments were approved by institutional animal ethics committee (IAEC) of NIPER-Hyderabad, in accordance with approved protocol. Animals were randomly divided into five groups (*n* = 12): (1) Control group, (2) BLM alone group (1 U/kg/day × 2), (3) BLM+WFA (2 mg/kg), (4) BLM+WFA (4 mg/kg), and (5) WFA alone (4 mg/kg). Mice were administered with 1 U/kg/day × 2 of BLM via oropharyngeal route while WFA was administered once daily from day 1 for 28 days by intraperitoneal (i.p.) route. To avoid pulmonary hemorrhage due to CO_2_ method of euthanasia, animals were euthanized using 5% Isoflurane (SurgiVet isoflurane vaporizer-4215061) before bronchoalveolar lavage (BAL) fluid collection. Lungs were harvested after euthanizing animals with CO_2_ asphyxiation on the day of termination for the pathological and molecular studies.

### Bronchoalveolar Lavage Fluid Parameters

Bronchoalveolar lavage fluid was collected thrice from each animal by passing 1 mL of ice-cold PBS into the trachea, followed by gentle aspiration of the fluid by inserting a catheter in the trachea (Bale et al., 2016). The percentage recovery of lavage fluid was approximately 85% and did not differ among the animal groups significantly. BAL fluid collected from each animal was pooled and subjected to total and differential cell count using automatic hematology Advia 2120i system (Siemens). Subsequently, BAL fluid was centrifuged at 300 *g*, for 10 min at 4°C, and the supernatant was stored at -80°C or used immediately to perform total protein content of BAL using Bradford assay and lactate dehydrogenase (LDH) levels were estimated using commercially available kit (Accurex, India).

### Lung to Body Weight Index

Animal body weights were recorded at the termination of the study. Lungs were collected after necropsy; cleaned and weighed. Lung weight index was calculated as ratio of lung weight to body weight.

### Lung Biochemical Assays

#### Estimation of Glutathione (GSH) Content

Ellman’s method was used to measure glutathione content in lung tissue supernatants (Moron et al., 1979). Lung tissues were homogenized using TRIS buffer and Ellman’s reagent [5, 5′-dithiobis-2-nitro benzoic acid (DTNB) solution] was added to the supernatants containing GSH buffer (125 mM potassium phosphate of pH 8.4), incubated for 5 min in dark and absorbance was measured at 412 nm using spectrophotometer (Spectramax M4, Molecular Devices, United States). Values obtained were compared with series of reduced glutathione standards (concentration range: 500 to 7.8 μM). Results were expressed as μM/mg of lung protein.

#### Estimation of Nitric Oxide Levels

Nitric oxide levels were estimated using Griess reagent (Guevara et al., 1998). Lung tissues were homogenized using TRIS buffer, supernatants were collected and mixed with equal proportions of Griess reagent, incubated in dark for 10 min and absorbance was measured at 548 nm without any delay. NO levels were expressed as μM/mg of lung protein with sodium nitrite taken as standard (concentration range: 100 to 1.5 μM).

#### Collagen Determination Using Hydroxyproline Assay

Assay for estimating hydroxyproline levels was performed to detect collagen levels as discussed (Limjunyawong et al., 2014) with slight modifications. Lung tissues were weighed, homogenized and subjected to acid hydrolysis. Oxidation of the tissues was done using Chloramine-T for 15 min followed by Ehrlich reagent addition. Samples were incubated at 60°C for 20 min, cooled and absorbance was read at 550 nm. Concentrations of samples were calculated using concentration-absorbance curve of hydroxyproline standards (concentration range: 125 to 3.9 μg/mL).

#### Sircol Assay for Collagen Estimation

Lung tissues were homogenized and supernatant was collected to which collagen binding dye was added and incubated for 1 h at 37°C, followed by centrifugation. Obtained visible red pellet was dissolved in 100% ethanol to remove excess dye and was again subjected to centrifugation. Subsequently, the pellet was dissolved in 0.5 M sodium chloride solution, incubated for 30 min at 37°C and absorbance was measured at 540 nm using spectrophotometer. Values were expressed as μM/mg normalized with lung protein content performed using Bradford assay.

### Pathological Investigations

Left lung tissues collected on the day of sacrifice were fixed in 10% non-buffered formalin solution and embedded in paraffin. Sections of 5 μm thickness were cut using microtome and subjected to H&E staining to observe pathologic morphological changes in the tissue. Further, to estimate the collagen deposition in the lung tissues, sections were stained with Masson’s trichrome and picrosirius red while the mast cell accumulation in lungs was estimated by toluidine blue staining as per the standard protocols (Veerappan et al., 2012; Godugu et al., 2013; Vogel et al., 2015).

### Quantification of TNF-α and IL-1β by ELISA

Lung tissues were weighed, homogenized, and subjected to centrifugation maintaining at 4°C. Tissue supernatants were stored at -80°C in aliquots or used immediately to estimate cytokine expression. Pro-inflammatory cytokines including TNF-α and IL-1β were evaluated for their expression in lung tissues using commercially available ELISA kits (eBioscience, TNF-α Catalogue No-50-173-67; IL-1β Catalogue No-50-112-8807).

### Immunohistochemistry

Lung tissue slides were deparaffinised in xylene and rehydrated in series of gradient alcohol. Antigen retrieval was performed by heating sections for 10 min in citrate buffer followed by eliminating endogenous peroxidase using 3% H_2_O_2_. The sections were incubated with 3% BSA to avoid non-specific background prior to incubation with primary antibodies including mouse anti-α-SMA antibody (1:100), rabbit anti-vimentin antibody (1:100), and rabbit anti-NF-κB p65 for 30 min at room temperature. HRP-linked polymer detection system was used to develop color reaction, sections were then counterstained with hematoxylin, mounted with resinous mounting solution and visualized for immunoreactivity and percentage of positive cells were calculated (Godugu et al., 2017).

### Western Blot Analysis

Procedure for western blot analysis was adopted from (Patel et al., 2015) with slight modifications. Briefly, cells were lysed using radio immunoprecipitation assay lysis buffer (RIPA) (Sigma-Aldrich) and lung tissues were homogenized using tissue protein extraction reagent (TEPER) lysis buffer, mixed with protease and phosphatase inhibitors (Sigma-Aldrich) maintained at 4°C, to obtain supernatants. Protein concentration was determined using Bicinchonic acid commercial assay kit (Sigma-Aldrich). Equal amounts of proteins were separated on SDS polyacrylamide gels and transferred onto polyvinyldifluoride (PVDF) membrane (Bio-Rad). After transfer, protein of interest was blocked for non-specific binding (3% BSA) and incubated with primary antibody overnight. Blots were washed thrice with TBST, each time for 10 min and incubated with HRP-conjugated secondary antibody for 1 h at room temperature. Membranes were visualized using enhanced chemiluminescence reagents by chemdoc (Vilber fusion Fx) and the protein bands were subjected to densitometry analysis using Image J software. The membranes were probed with β-actin antibody as an internal control. Each blot shown represents at least three independent experiments.

### Statistical Analysis

All results (*in vitro* and *in vivo*) were expressed as mean ± SEM of three independent experiments. Statistical analysis was performed by ANOVA followed by Tukey’s *post hoc* test. All statistical analyses were performed using GraphPad Prism^®^ Version 5 software. Probability values less than 0.05 levels were considered as statistically significant.

## Results

### Effect of WFA on Cell Viability and TGF-β1 Induced Cell Migration

**FIGURE 1:**
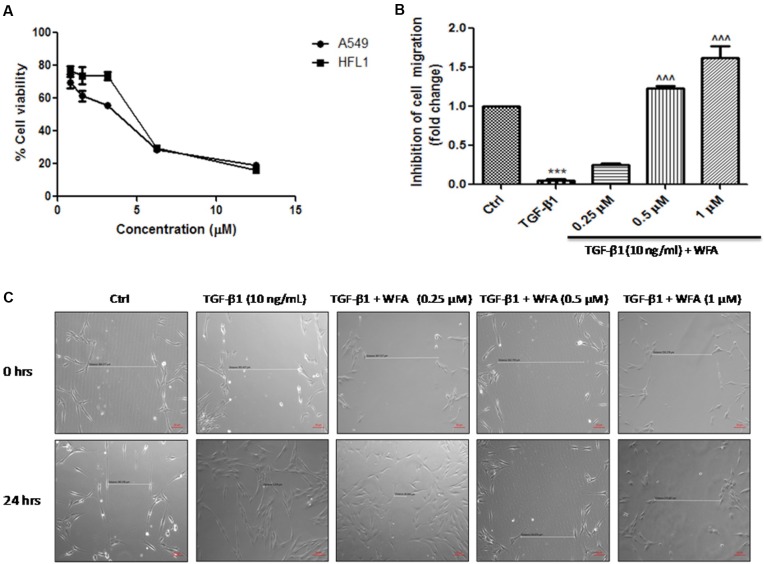
Effect of WFA on cell viability and migration after 24 h of TGF-β1 stimulation. **(A)** Effect of WFA on cell viability of A549 and HFL1 cell lines, **(B)** graphical representation denoting the effect of WFA on inhibition of cell migration of HFL1 cells analyzed using Image J software, and **(C)** representation of phase contrast images of cells depicting the role of WFA in inhibiting TGF-β1 induced cell migration. Statistical significance was tested using one way ANOVA, ^∗∗∗^*p* < 0.001 of TGF-β1 versus control; ˆ ˆ ˆ*p* < 0.001 of WFA with TGF-β1 versus TGF-β1 alone.

*In vitro* effects of WFA on cell viability were tested on A549 (human alveolar adenocarcinomic basal epithelial cells) and HFL1 (human fetal lung fibroblasts) cells; IC_50_ values were found to be 3.46 ± 0.2 and 4.45 ± 0.12 μM respectively at 24 h studied using MTT [3-(4, 5-dimethylythiazol-2-yl)-2, 5-diphenyl tetrazolium bromide] assay (**Figure 1A**). Based on the results of cell viability assay performed at various concentrations, maximum safe concentrations (<1 μM) were selected so that viability of the cells was not affected upon WFA treatment and concentration dependent studies were performed with WFA at 0.25, 0.5, and 1 μM in cultured cells. TGF-β1 stimulation promotes the rate of cellular migration (Barrett et al., 2017). Therefore, to delineate the effect of WFA on cell migration, wound scratch assay was performed with WFA at concentrations of 0.25, 0.5, and 1 μM on TGF-β1 (10 ng/mL) treatment for 24 h in HFL1 cells. WFA ameliorated TGF-β1 induced rate of cell migration (**Figures 1B,C**) in a concentration dependent manner demonstrating the decrease in the inducible cell proliferation rate by WFA. It was also evident that there was a significant decrease in migration rate of HFL1 cells by WFA at 0.5 and 1 μM, compared to control fibroblast cells suggesting the inhibitory effect of WFA on constitutive cell proliferation where in WFA attenuated the basal cell migration rate of fibroblasts.

### WFA Ameliorated the EMT Related Proteins on TGF-β1 Induction *in Vitro*

**FIGURE 2:**
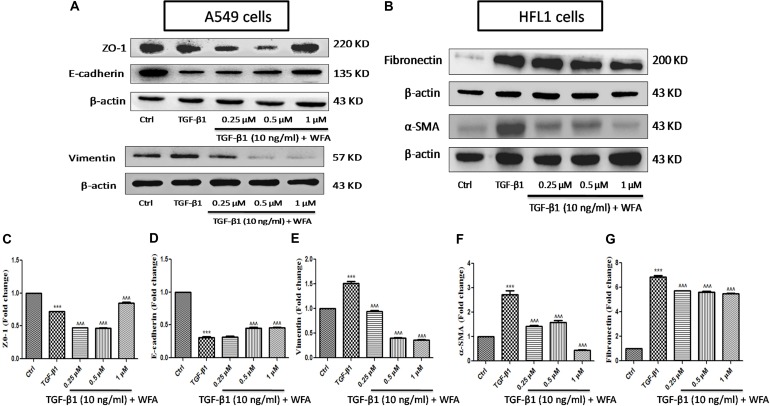
Effect of WFA on EMT related cell markers *in vitro* studied using western blot analysis. **(A)** A549 cells, **(B)** HFL1 cells, and **(C–G)** indicate graphical representation of quantified EMT protein expression using Image J software. **(C)** ZO-1, **(D)** E-cadherin, **(E)** Vimentin, **(F)** α-SMA, and **(G)** Fibronectin. Statistical significance was tested using one way ANOVA, ^∗∗∗^*p* < 0.001 of TGF-β1 versus control; ˆ ˆ ˆ*p* < 0.001 of WFA with TGF-β1 versus TGF-β1 alone.

EMT, the transformation of fibroblasts to myofibroblasts is crucial in pathogenesis of fibrotic disorders of different organs (Li et al., 2016). In *in vitro* studies, TGF-β1 is a key cytokine used to induce EMT events in various fibroblasts, specifically in A549 cells where EMT plays a prominent role. Therefore expression of some of the EMT related proteins were investigated upon WFA treatment on both A549 and HFL1 cell lines (**Figure 2**). TGF-β1 stimulated cells resulted in significant damping of both epithelial markers E-cadherin, Zonula occludens (ZO-1) and enhanced expression of vimentin. WFA treatment increased the expression of E-cadherin as well as ZO-1 and decreased the expression of mesenchymal vimentin in A549 cells concentration dependently.

**FIGURE 3:**
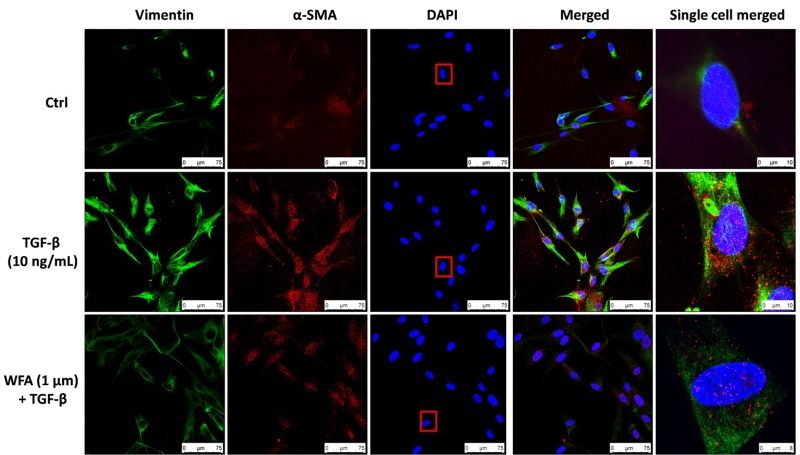
Immunofluoresence evidence for decreased expression of Vimentin and α-SMA proteins by WFA in lung fibroblasts. HFL1 cells were co-stained with antibodies against Vimentin (green-FITC) and α-SMA (red-rhodamine), while nuclei were stained with DAPI (blue). The overlay of two proteins and nuclei were shown as merged image. A single cell was randomly selected to significantly view the downregulation of these proteins by WFA on TGF-β1 stimulation as highlighted in a rectangular box of DAPI stain. Single cell merged represents the overlay of Vimentin and α-SMA expression along with DAPI of the selected cell. WFA at 1 μM concentration significantly reduced the expression of TGF-β1 stimulated Vimentin and α-SMA. Images were captured using confocal microscope (Leica TCS SP8 Laser Scanning Spectral Confocal).

Consistent with the effect of EMT inhibition of WFA on A549 cells, WFA also decreased the expression of key mesenchymal markers including α-smooth muscle actin (α-SMA) and fibronectin in TGF-β1 induced fibroblast cells in concentration dependent manner. To further confirm the effects of WFA on EMT markers, immunofluoresence assay was carried out on fibroblast cells (**Figure 3**). Results of immunocytochemistry assay clearly revealed the enhanced expression of mesenchymal markers α-SMA and vimentin in TGF-β1 treated cells compared to control cells. The distribution and expression of both α-SMA and vimentin were decreased upon WFA treatment (**Figure 3**) which was in consistency with the key findings of EMT events obtained by western blot.

### WFA Attenuated the Expression of Pro-fibrotic Proteins on TGF-β1 Induction in Lung Fibroblasts via Smad Signaling Cascade

**FIGURE 4:**
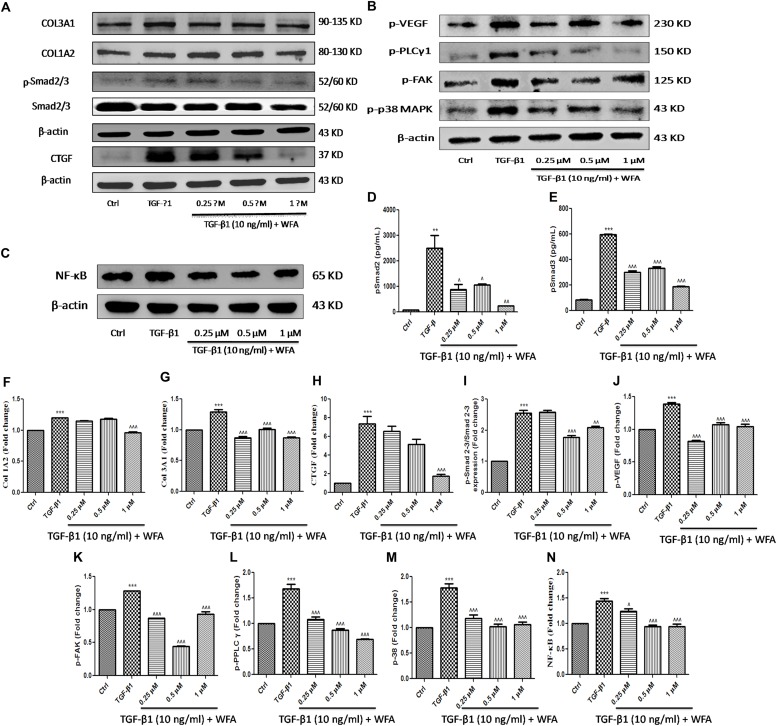
Effect of WFA on fibrotic, inflammation, and angiogenic proteins on HFL1 cells studied using immunoblot analysis. Representation of WFA on **(A)** fibrotic proteins, **(B)** angiogenic proteins, **(C)** NF-κB p65; **(D,E)** represents the effect of WFA on p-Smad 2 and p-Smad 3 proteins respectively analyzed by TGF-β bioplex analysis kit. **(F–N)** Indicate graphical representation of quantified protein expression. **(F)** Col 1A2, **(G)** Col 3A1, **(H)** CTGF, **(I)** p-Smad 2–3/Smad 2–3 expression, **(J)** p-VEGF, **(K)** p-FAK, **(L)** p-PPLC γ1, **(M)** p-38 MAPK, and **(N)** NF-κB p65. Statistical significance was tested using one way ANOVA, ^∗∗^*p* < 0.01, ^∗∗∗^*p* < 0.001 of TGF-β1 versus control; ˆ*p* < 0.05, ˆˆ*p* < 0.01, ˆ ˆ ˆ*p* < 0.001 of WFA with TGF-β1 versus TGF-β1 alone.

As WFA ameliorated EMT *in vitro* (A549 and HFL1), we further examined the molecular mechanism through which WFA suppresses TGF-β1 induced EMT. TGF-β1 is a key fibrotic cytokine that drives EMT by excess ECM synthesis (Moustakas and Heldin, 2016). TGF-β1 stimulation phosphorylates Smad 2/3 and forms heterocomplex with Smad 4 which modulates the target gene expression (Nakao et al., 1997). We assessed the effects of WFA on TGF-β1/Smad signaling pathway and as expected, there was a marked increase of p-Smad 2/3 protein expression in TGF-β1 treated cells compared to control cells and WFA significantly attenuated the phosphorylation of Smad 2/3 (**Figure 4A,I**).

Connective tissue growth factor (CTGF) is recognized as a major matricellular protein induced by TGF β1, functions as a central mediator in fibrosis was significantly increased in TGF-β1 treated cells and WFA decreased CTGF expression in a concentration dependent manner (**Figures 4A,H**). WFA effects on collagen 1A1 and collagen 3A2 were evaluated for their expression on TGF-β1 stimulation and found significant reduction in the expression of these proteins compared to TGF-β1 treated cells (**Figures 4F,G**). To confirm the anti-fibrotic effects of WFA mediated by Smad 2/3 signaling, *in vitro* fibroblasts cell lysates were investigated for their expression using TGF-β signaling 6-plex magnetic bead based kit (**Figures 4D,E**). Bioplex assay results implicated the amelioration of p-Smad 2 and p-Smad 3 proteins by WFA intervention on TGF-β1 stimulation in a concentration dependent manner which correlated with the results of p-Smad 2/3 expression obtained from western blot. These results demonstrated that WFA may attenuate PF through inhibiting TGF β1-Smad 2/3 signaling pathway.

### WFA Ameliorated TGF-β1 Driven Angiogenesis and Inflammation in Cultured Fibroblasts and Lung Tissue Sections

WFA is reported to exert anti-angiogenic effects in some cancers and to effectuate the role of WFA as anti-angiogenic agent in PF, some of the significant target proteins of angiogenesis like p-p38 MAPK (mitogen activated protein kinase), p-VEGF (vascular endothelial growth factor), p-FAK (focal adhesion kinase), and p-PLCγ1 (phospholipase C) were evaluated (**Figure 4B**). Notable increase in expression of the aforementioned proteins was observed in TGF-β1 treated HFL1 cells. WFA treatment resulted in significant decreased expression of p-p38 MAPK and its key effector molecule p-VEGF concentration dependently. Further, direct intracellular signaling molecules of VEGF including p-FAK and p-PLC γ1 were significantly attenuated by WFA, suggesting the possible role of WFA as anti-angiogenic agent in ameliorating PF. Consistent with this, p-VEGF expression in BLM treated lung sections was significantly ameliorated by WFA at both doses (**Figures 4J–M**). As inflammation is one of the critical phases in fibrotic progression, effect of WFA on key inflammatory mediator, NF-κB p65 was evaluated. WFA significantly decreased the enhanced expression of NF-κB p65 in a concentration dependent manner (**Figure 4C,N**).

### Preventive Effects of WFA on Lung Injury and Fibrosis in BLM Induced Murine Model

**FIGURE 5:**
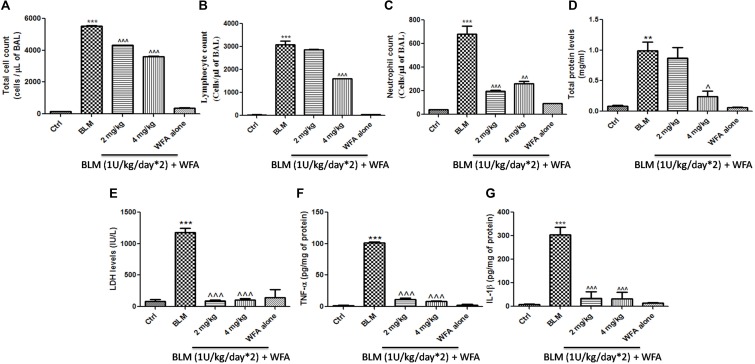
Effect of WFA on BALF cellular infiltrates and lung tissue cytokine expression. BALF obtained from the lungs was used for the measurement of **(A)** total cell count, **(B)** lymphocytes, **(C)** neutrophil count. WFA significantly reduced these infiltrations. BALF was centrifuged and supernatant was estimated for expression of **(D)** total protein content and **(E)** LDH levels. Decreased expression of **(F)** TNF-α and **(G)** IL-1β in lung tissue supernatants was observed with WFA treatment. Statistical significance was tested using one way ANOVA, ^∗∗^*p* < 0.01, ^∗∗∗^*p* < 0.001 of TGF-β 1 versus Ctrl; ˆ*p* < 0.05, ˆˆ*p* < 0.01, ˆˆˆ*p* < 0.001 of WFA with TGF-β1 versus TGF-β1 alone.

**FIGURE 6:**
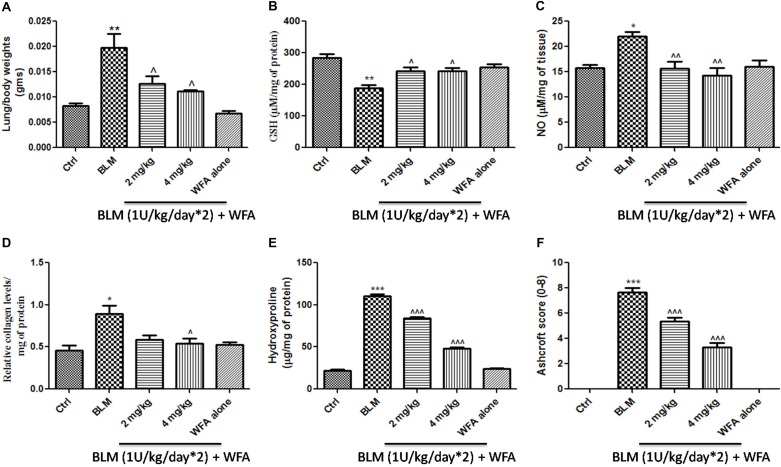
Effect of WFA on lung biochemical parameters and fibrotic markers. **(A)** Lung to body weight index was measured on the day of study termination; WFA treatment **(B)** elevated the levels of glutathione content and **(C)** reduced the expression of lung nitric oxide in BLM challenged mice. WFA inhibited the collagen expression evident from **(D)** sircol assay and **(E)** hydroxyproline assay. **(F)** Indicates the Ashcroft scoring of H&E stained lung tissues based on the progression of fibrosis. Statistical significance was tested using one way ANOVA, ^∗^*p* < 0.05, ^∗∗^*p* < 0.01, ^∗∗∗^*p* < 0.001 of TGF-β1 versus control; ˆ*p* < 0.05, ˆˆ*p* < 0.01, ˆ ˆ ˆ*p* < 0.001 of WFA with TGF-β1 versus TGF-β1 alone.

Withaferin A significantly decreased the total number of infiltrated cells in BALF which were elevated in BALF collected from BLM induced murine lungs (**Figure 5A**). Likewise, WFA significantly decreased the infiltrations of lymphocytes and neutrophils in a dose-dependent manner in BLM treated mice (**Figures 5B,C**). In parallel, WFA decreased the levels of BALF total protein content in BLM challenged mice, suggesting that WFA may attenuate lung microvascular leakage (**Figure 5D**). Furthermore, WFA also inhibited the lung damage induced by BLM illustrated by the decrease in LDH activity in BALF (**Figure 5E**). In addition to the recruitment of lymphocytes and neutrophils, the inflammatory phase of BLM is also featured by significant lung edema due to severe lung injury and ECM deposition, as measured by lung to body weight index which was substantially decreased by WFA (**Figure 6A**). Excessive collagen deposition in lung tissues of BLM treated group was reflected by increase in soluble collagen levels and hydroxyproline content respectively (**Figures 6D,E**). WFA reduced this BLM induced hydroxyproline and soluble collagen levels in a dose-dependent manner demonstrating the anti-fibrotic effect of WFA. Further, we examined the effect of WFA on surrogate parameters of oxidative and nitrative stress including glutathione levels and nitric oxide content respectively (**Figures 6B,C**). WFA augmented the expression of glutathione and decreased the nitric oxide levels in lung tissues.

### WFA Restored Histological Changes, Decreased Collagen Content, and Mast Cell Infiltration in Lungs of BLM Challenged Mice

**FIGURE 7:**
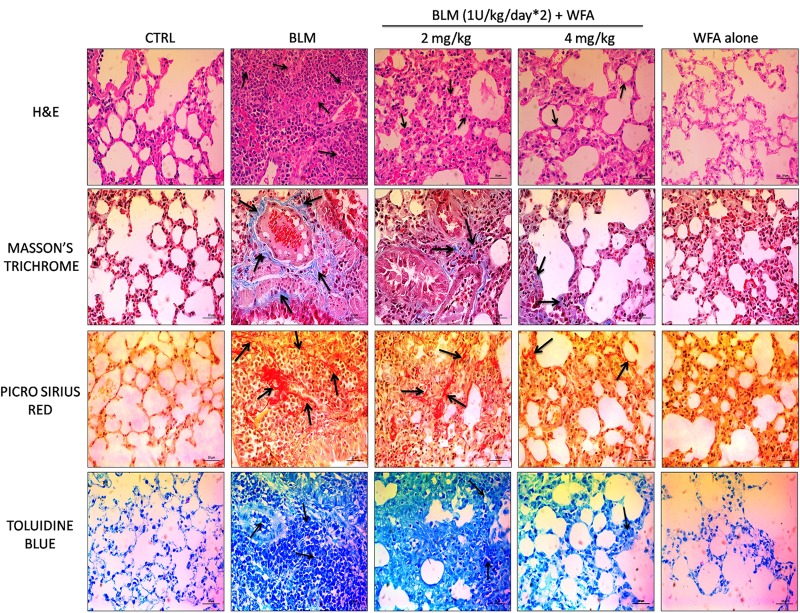
Effect of WFA on histological changes in lung tissues at 28 day post BLM induction. Photomicrographs exhibit staining of mice lung tissues with H&E, Masson’s trichrome, picrosirius red, and toluidine blue as shown from top to bottom. Arrows indicate the extent of pathological features of respective stains. WFA treated lung sections reduced the severity of lung damage as shown in H&E stained tissues, decreased collagen content as evident from blue and red color of Masson’s and picrosirius red stains respectively, and reduced the mast cell accumulation reflected from decreased blue color of WFA treated lung sections. Magnification: 400X.

To validate whether WFA exerts anti-fibrotic effects by modulating histological changes, we performed Hematoxylin and Eosin (H&E) staining to observe key morphological alveolar changes while Masson’s trichrome and picrosirius red stains were performed to evaluate collagen deposition and toluidine blue staining to detect extent of mast cell infiltration (**Figure 7**). A standard quantitative analysis based on improved Ashcroft scoring system was done in H&E stained sections to examine the histological changes (**Figure 6F**). H&E stained BLM treated lung tissues showed markedly increased alveolar thickness and obliteration of pulmonary musculature with fibrotic masses, while control lung tissues exhibited no fibrotic burden with normal lung architecture. WFA treatment at 2 mg/kg reduced the fibrous obliteration; excluding entailed single fibrotic masses while WFA at 4 mg/kg resulted in lung architecture devoid of fibrotic masses but with slightly enlarged alveoli and was comparable to normal lung architecture. In accordance with the observed restoration of histological changes, the anti-fibrotic effect of WFA was further supported by Masson’s trichrome and picrosirius red stains, in which WFA reduced the increased collagen deposition induced by BLM. Toluidine blue staining of lung sections illustrated decreased density of accumulated mast cell infiltration in WFA treated groups compared to BLM challenged mice.

### Anti-inflammatory, Anti-fibrotic, and EMT Inhibitory Effects of WFA *in Vivo*

**FIGURE 8:**
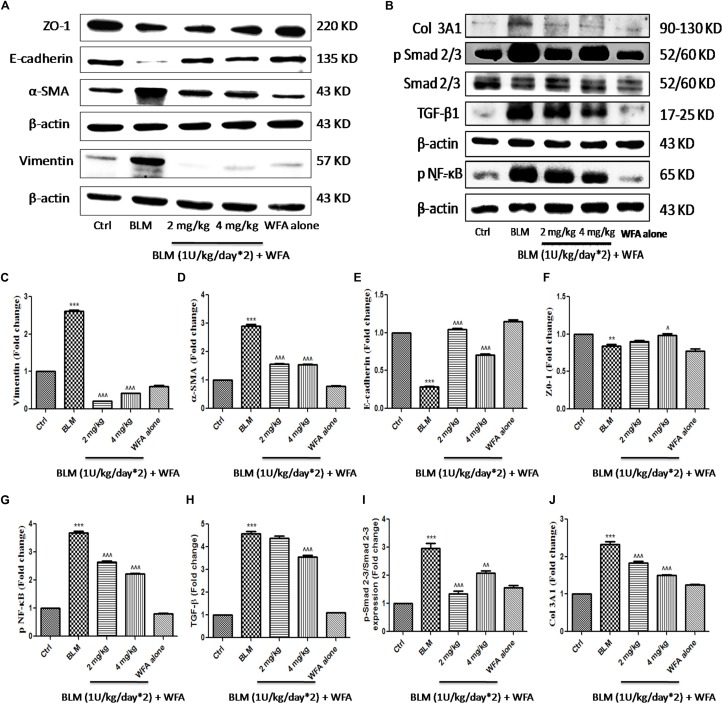
Effect of WFA on EMT and fibrotic proteins in BLM challenged mice studied using western blot analysis. Representation of WFA on **(A)** EMT related proteins, **(B)** fibrotic proteins; **(C–J)** indicates graphical representation of quantified protein expression. **(C)** Vimentin, **(D)** α-SMA, **(E)** E-cadherin, **(F)** ZO-1, **(G)** NF-κB p65, **(H)** TGF-β, **(I)** p-Smad 2–3/Smad 2–3 expression, and **(J)** COL 3A1. Statistical significance was tested using one way ANOVA, ^∗∗^*p* < 0.01, ^∗∗∗^*p* < 0.001 of TGF-β1 versus Ctrl; ˆ*p* < 0.05, ˆˆ*p* < 0.01, ˆ ˆ ˆ*p* < 0.001 of WFA with TGF-β1 versus TGF-β1 alone.

**FIGURE 9:**
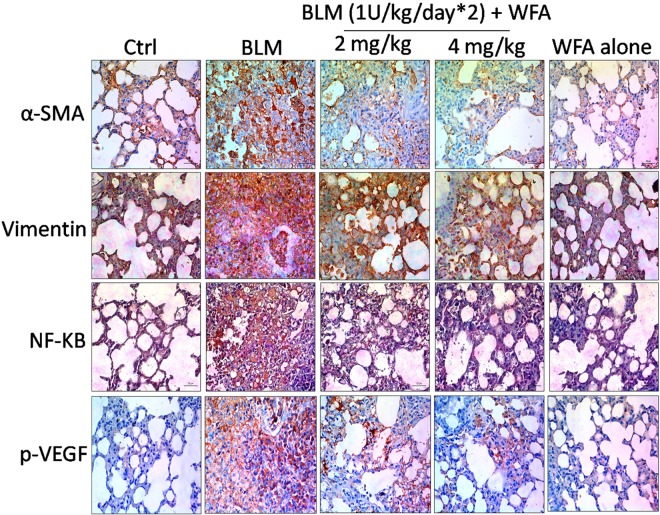
Effect of WFA on EMT, inflammatory and angiogenic proteins assayed using immunohistochemistry. Lung sections after processing were stained for antibodies against α-SMA, Vimentin, NF-κB p65, and p-VEGF proteins to evaluate the effect of WFA on expression of respective proteins. Significant elevated expression of all these proteins were observed in BLM challenged mice reflected from brown coloration and WFA substantially reduced the increased expression of proteins α-SMA, Vimentin, and NF-κB p65 in dose-dependent manner evidenced from decreased immunopositive cells on WFA treatment, while there was no considerable immunopositive cells in control and WFA alone groups. Magnification: 400X.

To investigate whether WFA modulates the production of inflammatory cytokines including IL-1β and TNF-α, we evaluated their expression by ELISA in lung tissue supernatants, which was ameliorated upon treatment with WFA (**Figures 5F,G**). Further, immunoblot assay revealed that WFA treatment decreased the expression of decisive inflammatory cytokine, NF-κB p65 in lung tissue. In addition, WFA dramatically ameliorated the EMT signaling matrix proteins and fibrotic events following BLM induction. WFA substantially increased the expression of epithelial marker E-cadherin, ZO-1 and decreased mesenchymal markers including α-SMA. Moreover, WFA attenuated the protein expression of pivotal fibrotic markers including TGF-β1, vimentin, collagen 3A1 and p-Smad 2/3 in dose-dependent manner, thus illustrating that the anti-fibrotic potential of WFA was mediated via TGF-β/Smad signaling (**Figure 8**). Next, we performed immunohistochemical staining for proteins α-SMA, vimentin, NF-κB p65, and p-VEGF in lung sections to measure the extent of myofibroblast transformation, inflammation, and angiogenesis, respectively (**Figure 9**). Immunostaining in various regions of the lung revealed significant decrease in the distribution and immunoreactivity of α-SMA, vimentin, and NF-κB p65 proteins by WFA dose-dependently. These results indicate that WFA bears the potential to inhibit inflammation, EMT, ECM, fibrotic responses, and angiogenesis in BLM challenged mice.

## Discussion

**FIGURE 10:**
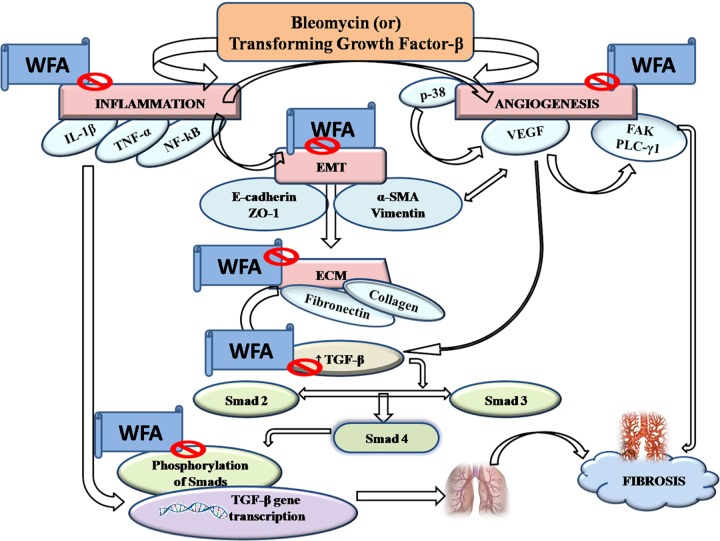
Graphical abstract: pictorially represents the amelioration of pulmonary fibrosis by Withaferin A via modulation of various signaling cascades.

Pulmonary fibrosis (PF) is defined as one of the serious kind of chronic and progressive fibrotic disorders of interstitial lung diseases of unknown etiology. Prevalence of PF is rapidly increasing ranging from 1.25 to 23.4 cases per 100,000 population in Europe, 16.3 to 17.4 per 100,000 population in United States and has turned into a major public health problem worldwide with a survival rate of less than 5 years (Ley et al., 2011; Nalysnyk et al., 2012; Lee et al., 2014). Unmet clinical needs provoke further research to identify and validate potential therapeutic targets and agents. In the present study, TGF-β1 induced *in vitro* and BLM induced *in vivo* fibrotic models were employed to evaluate the effect of WFA as an anti-fibrotic agent (Xu et al., 2007; Moeller et al., 2008). WFA, a steroidal lactone from Indian traditional herb, *Withania somnifera* is reported to cause disruption of vimentin intermediate filaments, thereby destabilizing the collagen mRNA that reflects its anti-fibrotic effect (Challa et al., 2012). This bioactive constituent also possesses potent anti-inflammatory activity by inhibiting key inflammatory cytokine NF-κB (Martorana et al., 2015). Thus the role of WFA as a therapeutic agent against PF involving the matricellular proteins, inflammatory mediators, and fibrotic markers at molecular level has been demonstrated in this study. We also investigated the possible role of WFA on TGF-β1 induced angiogenesis in cultured fibroblasts and *in vivo* (**Figure 10**).

Initially, we investigated the effects of WFA on cell viability on A549 and HFL1 cells and based on the results, 0.25, 0.5, and 1 μM concentrations of WFA were selected for further molecular studies. Since PF is a result of uncontrolled wound healing, ability of the cells to migrate and proliferate is considered as initial stage of wound repair (Betensley et al., 2016). Effect of WFA on cellular migration was performed and results of the study demonstrated that WFA significantly blocked the proliferation of fibroblasts as evident from wider wound gap in WFA treated cells as compared to control cells, which is consistent with the reported studies of WFA in colon cancer HCT116 cells (Choi and Kim, 2015). Mounting evidence suggest that A549 (lung type-II alveolar epithelial) cells undergo obvious EMT transformation and play a crucial role in pathogenesis of PF; accompanied by increased expression of mesenchymal markers and decreased expression of epithelial markers that results in excess ECM deposition (Özbek et al., 2010). Further lung matrix homeostasis and whole fibrotic events can be well-assayed in HFL1 cells, where TGF-β1 prominently stimulates myofibroblast formation (Clark et al., 1997). Therefore, we explored EMT inhibition properties in A549 and HFL1 cells due to predominant EMT occurrence in both the cell lines, but lung matrix homeostasis and whole fibrotic events can be well-assayed only in HFL1 cells, thus anti-fibrotic events of Withaferin A was further investigated only in HFL1 cells in the present study.

During EMT progression, a typical epithelial marker E-cadherin is repressed and switches to expression of N-cadherin (Maeda et al., 2005). Similarly, another important epithelial cell marker is ZO-1, a tight junction membrane associated protein whose expression is decreased during the EMT progression and is considered as a reliable indicator of EMT (Reichert et al., 2000). We evaluated this possibility and found that expression of both epithelial markers E-cadherin and ZO-1 to be remarkably decreased upon TGF-β1 stimulation and WFA restored the decreased levels of these proteins in concentration dependent manner. Consistently, increased E-cadherin and ZO-1 expression by WFA in BLM challenged mice was observed in this study. By contrast, α-SMA and vimentin are mesenchymal biomarkers whose expression is aberrantly increased as EMT progresses (Kokkinos et al., 2007; Ding et al., 2014). Vimentin is one of the cytoskeleton proteins that regulate functions of a plethora of cellular and biochemical processes and propagates EMT together with actin. Furthermore vimentin stabilizes collagen mRNA expression and aids in excess collagen deposition (Challa et al., 2012). The role of WFA as a vimentin inhibitor has been documented in several studies and was therefore evaluated for its effect on vimentin down-regulation in PF. As expected, protein expression of α-SMA and vimentin was significantly increased in TGF-β1 stimulated HFL1 cells and WFA mitigated this effect. In support of this, immunofluoresence assay of these proteins performed in HFL1 cells revealed their decreased expression on treatment with WFA. Moreover, we have investigated the expression of α-SMA and vimentin in lung tissues of BLM induced mice and the results revealed a significant dose-dependent decrease in the expression of these proteins by WFA. Further to confirm their expression in lung tissues, immunohistochemistry was performed and a notable decreased expression of α-SMA and vimentin by WFA was observed as evident by their decreased immunopositivity. Overall the findings of immunoblotting and immunohistochemistry of α-SMA and vimentin suggest the potential of EMT inhibition in PF by WFA. Further, the protective effect of EMT inhibition by WFA was confirmed using immunofluoresence assay performed on proteins α-SMA and vimentin using confocal microscope.

During the course of fibrosis, the subsequent step of EMT is ECM deposition. Fibronectin, a ubiquitous glycoprotein of ECM that plays a role in tissue remodeling is considered as a decisive contributing factor to switch from normal wound healing to deregulated fibrosis (Petrini et al., 2017). Excess fibronectin is produced from endothelial cells and fibroblasts upon stimulation of growth factors including platelet derived growth factor and TGF-β1 (Hocevar et al., 1999; Zhao et al., 2017). Hence, the role of WFA on fibronectin was evaluated and we observed dramatic decrease in expression of fibronectin in WFA treated HFL1 cells. CTGF is another matricellular protein that enables persistent fibrosis by triggering EMT, facilitates ECM deposition and remodeling (Weston et al., 2003). Further, several lines of evidence reported that increased CTGF expression contributes to elevated α-SMA expression that are implicated in PF (Zhang et al., 2004). The present study therefore evaluated the effect of WFA on CTGF and significant decreased expression of CTGF was observed on WFA treated TGF-β1 stimulated cells in concentration dependent manner. Additionally, the role of collagen, the most widely expressed protein of connective tissue as a pivotal ECM component has been demonstrated in several studies (Di Lullo et al., 2002). Collagen 1 and collagen 3 dependent cellular processes are found to be detrimental in PF (Hansen et al., 2016). It is well-documented from a recent study that TGF-β1 activated CTGF perhaps enhances the expression of collagen 1 mediated Smad 3 signaling (Cheng et al., 2015). As a result, collagen expression on WFA treatment both *in vitro* and *in vivo* was investigated using a series of experiments for further confirmation. Both collagen 1A2 and collagen 3A1 levels were substantially decreased by WFA in TGF-β1 stimulated cells. Further, results of hydroxyproline and sircol assay evidenced the reduced collagen content in lung tissues of BLM challenged mice by WFA which were consistent with the results of Masson’s trichrome and picrosirius red stained lung sections respectively, wherein WFA ameliorated collagen levels in dose-dependent manner. In addition, immunoblotting analysis of lung tissues also revealed reduced collagen 3A1 expression upon WFA treatment.

The development of fibrotic events is possible due to deregulation of several signaling cascades including Smad dependent and independent pathways (Derynck and Zhang, 2003). Aberrant TGF-β1 activation has been reported in several studies of BLM induced model of PF (Khalil et al., 2002; Zhao et al., 2002). As speculated, TGF-β1 expression was significantly elevated in BLM induced mice and a subsequent decreased TGF-β1 expression was observed in lung tissues treated with WFA. Moreover, intracellular phosphorylation of Smad 2/3 is a major determinant in mediating TGF-β1 induced fibrotic events (Evans et al., 2003; Gu et al., 2007; Walton et al., 2017). The present study is aimed at investigating the potential of WFA in suppressing Smad activation. In context with the established reports, immunoblot and bioplex analysis results of the study exhibited significant increase of p-Smad 2/3 expression in TGF-β1 stimulated cells and BLM induced lung tissues while, WFA significantly ameliorated the phosphorylation of Smad 2/3. Overall, these findings revealed WFA as a potential inhibitor of TGF-β1 mediated Smad signaling cascade in attenuating PF.

Toward the direction of oxidative and nitrative stress in PF, WFA augmented the levels of glutathione content and curbed down nitric oxide levels in lung tissues. Extensive influx of inflammatory cells particularly lymphocytes and neutrophils in BALF of oropharyngeally aspirated BLM mice was observed, as reported in previous studies (Karpel et al., 1989; Obayashi et al., 1997). WFA reduced pulmonary inflammatory cytological infiltrates of BALF (lymphocytes and neutrophils) following BLM aspiration and restored the expression of LDH and total protein content, the key inflammatory biomarkers of PF (Newton et al., 2017). In agreement with the attenuation of cellular infiltrates by WFA, lung tissue infiltration was also reduced by WFA as evident from pathological findings featured by reduced ECM deposition from decreased alveolar septal thickening. Besides BAL cell infiltrations, mast cell deposition in lung tissues aggravates PF and WFA significantly diminished mast cell accumulation as reflected by toluidine blue staining (Wygrecka et al., 2013). Further, inflammatory cytokines including IL-1β, TNF-α, and NF-κB leave no gap in triggering fibrotic events (Wilson et al., 2010; Hou et al., 2018). Moreover, WFA is a profound inhibitor of inflammatory responses in both cellular and animal models and thus was investigated in the present study and was found to alleviate the expression of those proteins in lung tissues upon WFA treatment. WFA as a potent inhibitor of NF-κB in T-cell mediated adaptive immune reactions prompted us to investigate its role on NF-κB in PF (Maitra et al., 2009). Remarkable decrease in expression of NF-κB p65 was observed in HFL1 cells and lung tissues from immunoblot studies. Further, immunohistochemistry results of NF-κB p65 protein confirmed the potential of WFA in down regulating inflammation associated with PF.

Angiogenesis plays a pivotal role in PF as demonstrated by elevated levels of angiogenenic chemokines in both experimental animal models and tissue specimens collected from IPF patients (Hanumegowda et al., 2012; Ziora et al., 2015). Further, it has been strongly revealed that VEGF, an angiogenic promoter is aberrantly expressed in lung specimens of PF patients suggesting angiogenesis as one of the prime factors in PF pathogenesis (Richter et al., 2005). TGF-β1 directly activates the pivotal angiogenic growth factor VEGF and also stimulates VEGF activation by various down streaming signaling mediators including α-SMA, fibronectin, and CTGF (Ferrari et al., 2009; Liu et al., 2014). Moreover, inflammatory cytokines including NF-κB and p38 MAPK also aggravates angiogenesis by phosphorylating the key effector molecule VEGF (Ye and Yuan, 2007; Kim et al., 2014). FAK, a cytoplasmic non-receptor tyrosine kinase protein is found to play a role in myofibroblasts differentiation and thus propagates angiogenesis mediated PF (Zhao and Guan, 2011). FAK inhibition attenuated EMT process in a study of BLM induced PF (Lagares et al., 2012). Further, PLC-γ1 is a downstream mediator of VEGF that is over expressed in angiogenesis (Lawson et al., 2003). It was inferred from the results of current study that WFA substantially reduced the expression of p-p38 MAPK, p-VEGF, p-FAK, and p-PLCγ1 proteins demonstrating the potential of WFA in attenuating angiogenesis driven PF. Taken together, the present study throws light on the significance of interplay of various pro-fibrotic and cellular matrix proteins, pro-inflammatory cytokines, and angiogenic factors in pathogenesis of PF and emphasizes on targeting such integral cellular processes for heading toward development of effective therapeutic interventions. WFA could possibly fit into this role of mitigating PF by inhibiting EMT and ECM progression, inflammation, and angiogenesis. However, further decisive studies are requisite for establishing WFA as a promising therapeutic intervention for treatment of PF.

## Author Contributions

CG and SB designed the research work. SB performed the research with PV and MS. SB and CG wrote the manuscript. All authors reviewed the final version of the manuscript.

## Conflict of Interest Statement

The authors declare that the research was conducted in the absence of any commercial or financial relationships that could be construed as a potential conflict of interest.

## Abbreviations

BLM: Bleomycin
ECM: extracellular matrix
EMT: epithelial to mesenchymal transition
PF: pulmonary fibrosis
TGF-β: transforming growth factor-β
WFA: Withaferin A
